# Utilization of cancer immunotherapy in sub-Saharan Africa

**DOI:** 10.3389/fonc.2023.1266514

**Published:** 2023-12-21

**Authors:** Elizabeth Olatunji, Saloni Patel, Katy Graef, Adedayo Joseph, Nwamaka Lasebikan, Abba Mallum, Chinelo Chigbo, Elizabeth Jaffee, Wil Ngwa

**Affiliations:** ^1^ Johns Hopkins University School of Medicine, Baltimore, MD, United States; ^2^ BIO Ventures for Global Health, Seattle, WA, United States; ^3^ Nigeria Sovereign Investment Authority-Lagos University Teaching Hospital (NSIA-LUTH) Cancer Center, Lagos University Teaching Hospital, Lagos, Nigeria; ^4^ Oncology Center, University of Nigeria Teaching Hospital Enugu, Enugu, Nigeria; ^5^ Department of Radiotherapy and Oncology, University of KwaZulu-Natal, Durban, South Africa; ^6^ Department of Oncology, Inkosi Albert Luthuli Central Hospital, Durban, South Africa; ^7^ Sidney Kimmel Comprehensive Cancer Center, Johns Hopkins, Baltimore, MD, United States; ^8^ Brigham and Women’s Hospital, Dana-Farber Cancer Institute, Harvard Medical School, Boston, MA, United States

**Keywords:** immunotherapy, sub-Saharan Africa, global oncology, cancer, immunotherapy training

## Abstract

**Introduction:**

The Lancet Oncology Commission for sub-Saharan Africa (SSA) predicts that cancer deaths will double from 520,158 per year to more than 1 million per year by the year 2040. These striking figures indicate a need to urgently evaluate cancer treatment infrastructure and resources in the region. Studies have found immunotherapy to be effective for the treatment of advanced-stage cancer, which almost 70% of patients in SSA present with. Despite immunotherapy’s significant therapeutic potential, its utilization in SSA is not well documented. The purpose of this study was to evaluate the landscape of immunotherapy in SSA.

**Methods:**

A Qualtrics survey assessing the existing infrastructure and training for safe immunotherapy administration was developed and distributed online via email and WhatsApp to 3,231 healthcare providers across SSA, with a target audience of healthcare providers serving patients with cancer. The survey contained 22 questions evaluating the accessibility, use, knowledge, and training on immunotherapy in SSA. Responses were collected between January and February 2023. Microsoft Excel was used to summarize and visually present the distribution of responses as counts and proportions.

**Results:**

292 responses were included from 28 countries in SSA. 29% of all respondents indicated their clinic has easy access to cancer immunotherapy and 46% indicated their clinic currently practices it. Of clinics that practiced immunotherapy (n = 133), 12% used genomic sequencing to assess the tumor mutational burden biomarker, and 44% assessed expression of the PD-L1 biomarker prior to immunotherapy administration. 46% of all respondents were familiar with immunotherapy. 11% indicated being adequately trained to administer it. Of these (n=33), 52% indicated also being trained to manage immune-related adverse events related to immunotherapy administration.

**Conclusion:**

Immunotherapy utilization and training is low in SSA and insufficient for the rising cancer burden. Increased accessibility and usage of biomarker testing to predict immunotherapy response, incorporation of immunotherapy training into continuous medical education, and increased access to immunotherapy drugs may be prerequisites for expanded utilization of immunotherapy in SSA.

## 1 Introduction

Recent years have seen the rise of cancer in sub-Saharan Africa (SSA). There were 801,392 new cases and 520,158 deaths in the region in 2020 (1), and experts predict a doubling of this incidence and mortality by 2040 without appropriate intervention, meaning over one million lives lost to cancer in SSA in 2040 (2, 3). Cancer deaths rates in SSA have exceeded the death rates of malaria, tuberculosis and AIDS combined (4). This rising cancer burden in SSA has been attributed to factors such as lifestyle changes, increasing life expectancy, infection, and the low priority of cancer in many healthcare systems in SSA (5). Cancer survival rates can provide estimates on the effectiveness of cancer care in a region (6). Countries in SSA have lower cancer survival rates than Western countries. The 5-year survival rate of women with breast cancer ranges from 85-90% in North America, Australia, Japan and Northern Europe, whereas it is 46% in Uganda, 39% in Algeria and 12% in The Gambia (7). This stark difference is likely attributable to limited awareness of cancer, cultural stigma, and lack of access to screening programs and appropriate treatment in SSA. The high mortality burden and the increasing threat of cancer in SSA warrants an assessment of the existing cancer treatment infrastructure and resources in the region.

Types of cancer treatment include surgery, chemotherapy, radiation therapy and immunotherapy. Surgical cancer treatment involves surgical excision of solid tumors. Chemotherapy involves medications that kill cancer cells, most commonly by damaging cancer cell DNA and preventing the cell from dividing (8). Radiation therapy uses high-energy ionizing radiation to damage cancer cell DNA and similarly, prevent them from dividing (9). Surgery, chemotherapy and radiation therapy have been used to treat cancer in SSA for years, but access to these resources has been limited by lack of trained personnel, facilities, and high medication costs (5, 10). Immunotherapy is a novel form of cancer treatment that aims to strengthen the body’s own immune system to control and eliminate cancer (11). This is distinct from other cancer treatments which directly target the cancer itself. Types of immunotherapy include monoclonal antibodies, checkpoint inhibitors, cytokines, CAR T-cell therapy and cancer vaccines (12), and while they all aim to strengthen immune function, their mechanisms of action vary. Immunotherapy may be used as a monotherapy, or in combination with other treatment modalities such as chemotherapy (13).

Existing studies have found immunotherapy to be efficacious for the curative treatment of some advanced cancers, including advanced prostate, lung, and urothelial cancers (14–16). The recent KEYNOTE trial showed durable antitumor activity of immunotherapy drugs pembrolizumab and lenvatinib for previously untreated advanced non-clear-cell renal cell carcinoma, promoting the use of this immunotherapy combination as a first-line treatment for that indication (17). The recent CheckMate 227 trial showed long-term efficacy (4 years minimum follow up) of first-line immunotherapy regimen nivolumab plus ipilimumab in patients with advanced non-small cell lung cancer (18). These findings may be significant in the context of SSA, where most cancer patients present with advanced stage disease, reducing the options for, and effectiveness of, cancer treatment (19, 20). The International Agency for Research on Cancer reports that 50-90% of women with breast cancer in SSA are diagnosed with late-stage, non-localized disease (21). It is estimated that up to 80% of cancer patients in Africa present to the hospital with late stage cancer, when the options for treatment are narrow (22). While it is important to introduce and reinforce interventions that aim to prevent this late presentation of disease in SSA, treatments effective against advanced cancer are also important, as they could contribute to the reduction of the high cancer mortality in the region.

Despite the strong curative potential of immunotherapy, it does not elicit a positive treatment response for all patients who receive it. Immunotherapy can produce a severe, uncontrolled autoimmune inflammatory response in recipients (23). Tumors can also create mutations or utilize other mechanisms to decrease the effectiveness of the treatment (24). For this reason, biomarkers are used to characterize a tumor and predict a patient’s response to immunotherapy. Biomarkers commonly used for this purpose are tumor mutational burden and PD-L1 testing. Tumor mutational burden is assessed by genome sequencing and quantifies the number of genetic mutations within a tumor (25). Quantification of mutations found that 10 mutations per megabase can predict an individual’s response to anti-PD-1 immunotherapy drugs such as pembrolizumab, nivolumab, and cemiplimab (26, 27). High tumor mutational burden has been correlated with a more effective anticancer response to immunotherapy. The American Food and Drug Association has even approved the use of immunotherapy such as pembrolizumab, for high tumor mutational burden indications, based on clinical trials demonstrating significantly increased efficacy of the therapy in patients with this biomarker (28, 29). PD-L1 is another biomarker used to predict a patient’s response to immunotherapy. A PD-L1 test assesses the presence of protein PD-L1 on the surface of cancer cells (30). Cancer cells with high quantities of PD-L1 can stop or slow down an individual’s immune response to the cancer, but immunotherapy drugs that target PD-L1 can augment an individual’s anticancer immune response. The presence of PD-L1 in tumor tissues has been found to correlate with a better clinical response to immunotherapy drugs targeting the PD-1/PD-L1 axis and better prognosis for certain cancers including metastatic melanoma, non-small cell lung cancer, and metastatic triple-negative breast cancer (31, 32). By using biomarkers such as tumor mutational burden and PD-L1 to predict response to and prognosis following immunotherapy, healthcare providers can reduce patient treatment costs and spare patients from unnecessary autoimmune side-effects.

Cancer immunotherapy may increase the curative effect of cancer treatment in SSA, due to its observed efficacy against advanced stage cancer. However, the utilization of immunotherapy has not been documented in the region. As such, this study aimed to assess SSA healthcare provider familiarity with, training in, and usage of, immunotherapy, to inform future research and policy aimed at combating the surging cancer incidence and mortality in SSA.

## 2 Materials and methods

A Qualtrics survey was developed and the survey link (**Supplementary Material 1**, https://jhmi.co1.qualtrics.com/jfe/form/SV_0IirBglMrRpAYbc) distributed online via email and WhatsApp to the SSA networks of the HypoAfrica clinical trial (33) and BIO Ventures for Global Health, which both include healthcare providers in SSA serving patients with cancer (34). This distribution targeted roughly 3,231 individuals across SSA who could choose to participate in the survey. Responses were collected between January 2023 and February 2023. The survey had 22 questions falling into one of two categories: general respondent characteristics (five questions) and immunotherapy utilization (17 questions). The target audience for the survey was healthcare providers in SSA serving patients with cancer. All respondents gave online written consent prior to answering the survey questions. Questions in the general respondent characteristics section assessed respondents’ clinic location, clinic type, position in their clinic, and interest and prior participation in clinical trials. These questions aimed to capture the distribution of and variation within the survey respondents and respondent interest in participating in future clinical trials. Questions on immunotherapy utilization assessed the current use of immunotherapy in respondent clinics and respondent knowledge of, and training for, immunotherapy administration. These questions aimed to capture details on immunotherapy use in SSA clinics, from general questions about availability of immunotherapy, to more specific questions about the types of immunotherapy used, the types of cancers targeted with immunotherapy, and access to biomarker testing. Most questions had a multiple-choice answer format. Five clinical and medical oncologists from the United States and Nigeria who are knowledgeable about cancer immunotherapy reviewed and gave feedback for adaptation of the survey prior to its distribution, to strengthen the survey’s capacity to measure the questions being assessed. These experts were a medical oncologist and professor from the Sidney Kimmel Comprehensive Cancer Center at Johns Hopkins, a clinical oncologist and lecturer from the University of Lagos College of Medicine, a clinical oncologist and research program director at the Nigeria Sovereign Investment Authority-Lagos University Teaching Hospital Cancer Center, the president of the Association of Radiation and Clinical Oncologists of Nigeria, and a clinical oncologist at the University of Nigeria Teaching Hospital Oncology Center. The survey was IRB approved by the University of Massachusetts Lowell Institutional Review Board and administered in English alone. Since the majority of the collected data were nominal, Microsoft Excel alone was used to summarize and visually present the distribution of responses as counts and proportions.

## 3 Results

### 3.1 Respondent characteristics

294 unique responses were obtained for a response rate of 9%. 2 survey responses were excluded as the respondents’ locations were based outside of SSA (i.e., Pakistan and the United Kingdom). 292 responses were received from 28 countries in SSA and were included in the analysis. Missing data were not imputed. **Table 1** shows the distribution of responses by country. The country from which most responses were obtained was Nigeria (n = 117, 40%). The countries from which the least responses were obtained were Mali, Senegal, Burkina Faso, Eswatini, Botswana, Mauritius, and Chad (n = 1 each, 0%). 51% of respondents worked at government-owned clinics, 32% at tertiary clinics, and 9% at private clinics (**Table 2**). 24% of respondents identified as nurses, 18% as clinical oncologists (i.e., trained in both medical and radiation oncology), 6% as radiation oncologists, and 5% as medical oncologists.

**Table 1:** Number of survey responses by country; n = 292.

Respondent Characteristics	n (%)
**Country ** Cameroon Nigeria Niger Kenya Ghana Tanzania Ethiopia Democratic Republic of the Congo Benin Uganda Ivory coast Mali Senegal Zambia Lesotho Togo Sudan Zimbabwe Rwanda Burundi Malawi Congo Republic Burkina Faso Eswatini South Africa Botswana Mauritius Chad Unknown	20 (7) 117 (40) 2 (1) 28 (11) 16 (5) 14 (5) 16 (5) 7 (2) 2 (1) 7 (2) 6 (2) 1 (0) 1 (0) 11 (4) 5 (2) 2 (1) 3 (1) 2 (1) 2 (1) 2 (1) 7 (2) 2 (1) 1 (0) 1 (0) 2 (1) 1 (0) 1 (0) 1 (0) 12 (4)

**Table 2:** Clinic type and healthcare position of all survey respondents; n = 292.

Respondent Characteristics	n (%)
**Clinic type ** Government owned Tertiary Private Other **Position ** Clinical oncologist Radiation oncologist Medical oncologist Surgical oncologist Medical physicist Nurse Other	148 (51) 95 (32) 26 (9) 23 (8) 55 (18) 18 (6) 16 (5) 7 (2) 9 (3) 74 (24) 126 (42)

### 3.2 Immunotherapy utilization and accessibility

**Figure 1:**
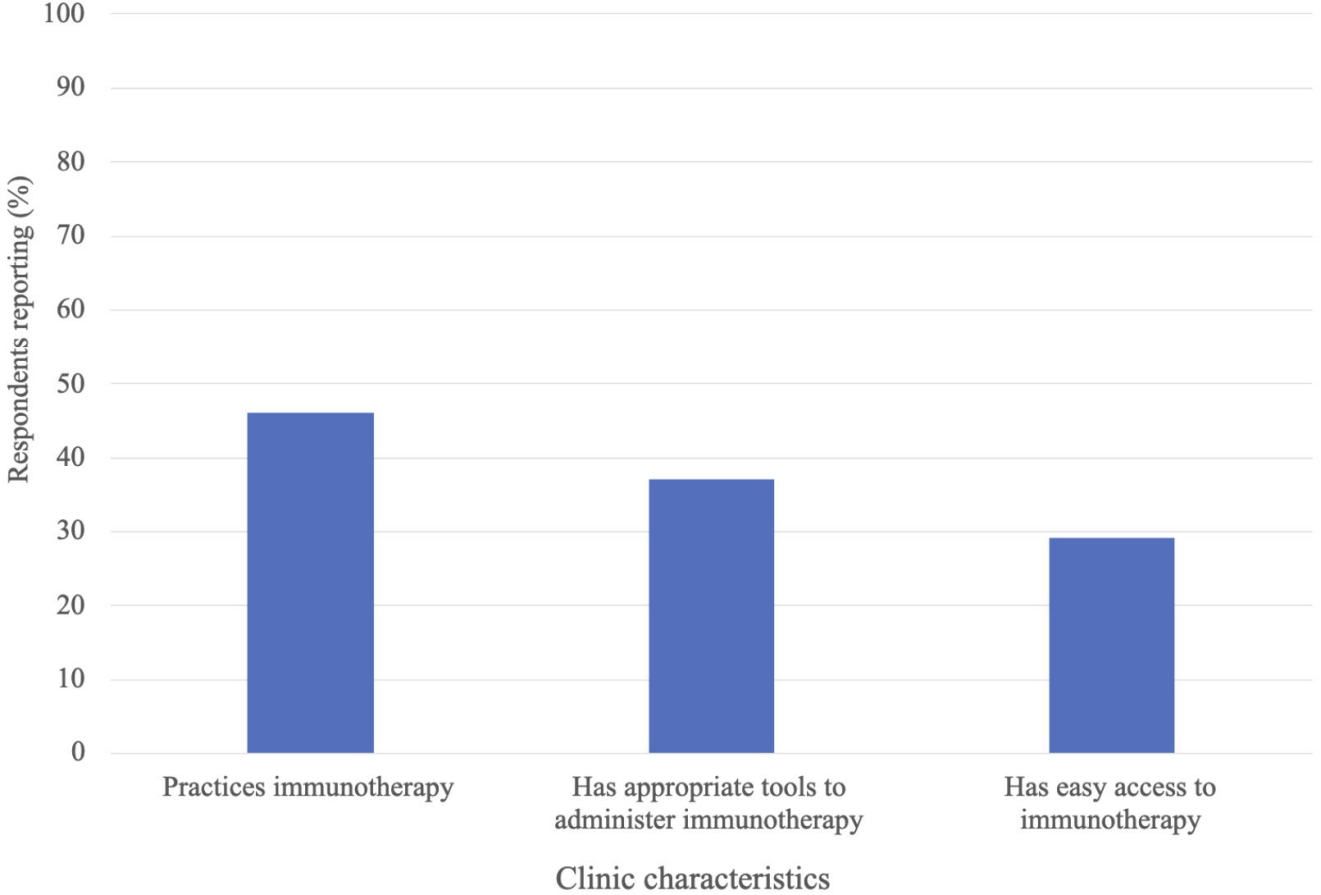
Immunotherapy utilization characteristics from all survey respondents’ clinics: n=292.

**Figure 2:**
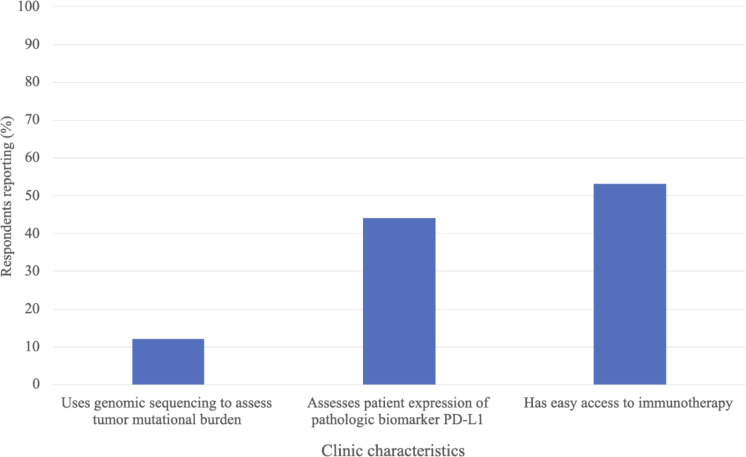
Immunotherapy predictive biomarker testing and accessibility at clinics where respondents reported current immunotherapy utilization, n=133.

**Figure 1** depicts the immunotherapy utilization and accessibility at respondents’ clinics. 46% of respondents reported that their clinic practices immunotherapy. 37% indicated that their clinic has the necessary tools to administer immunotherapy (i.e., storage, formulation, infusion tools), and 29% indicated having easy access to immunotherapy through drug companies, independent providers, their healthcare system, or their government. Of the respondents indicating their clinic practices immunotherapy (n = 133), 12% indicated their clinic uses genomic sequencing to assess tumor mutational burden prior to immunotherapy administration, 44% indicated that their clinic conducts PD-L1 testing prior to immunotherapy administration (testing occurs either within the clinic or at an external site) and 53% reported their clinic having easy access to immunotherapy (**Figure 2**). The most common immunotherapy available for administration at the sites practicing immunotherapy were monoclonal antibodies (71%), checkpoint inhibitors (29%) and cytokines (13%; **Table 3**). The most common primary cancers for which immunotherapy was used at the sites practicing immunotherapy were breast cancer (67%), lymphoma (39%), prostate cancer (29%), lung cancer (28%) and liver cancer (28%).

**Table 3:** Immunotherapy usage and cancer targets at clinics where respondents reported current immunotherapy utilization, n = 133.

Respondent Characteristics	n (%)
**Immunotherapy available for administration ** Monoclonal antibodies Checkpoint inhibitors Cytokines Cancer vaccines CAR T-cell therapy None of the above Unsure Other **Primary cancers where immunotherapy is used for treatment ** Head and Neck Cancers Spinal Cancer Breast Cancer Lung Cancer Liver Cancer Pancreatic Cancer Prostate Cancer Uterine Cancer Cervical Cancer Rectal Cancer Bladder Cancer Soft Tissue Sarcoma Lymphoma Leukemia None of the above Unsure Other	95 (71) 38 (29) 30 (23) 18 (14) 6 (5) 7 (5) 19 (14) 3 (2) 29 (22) 8 (6) 89 (67) 37 (28) 37 (28) 21 (16) 38 (29) 19 (14) 28 (21) 29 (22) 17 (13) 23 (17) 52 (39) 31 (23) 1 (0) 6 (5) 11 (8)

### 3.3 Immunotherapy knowledge and training

**Figure 3:**
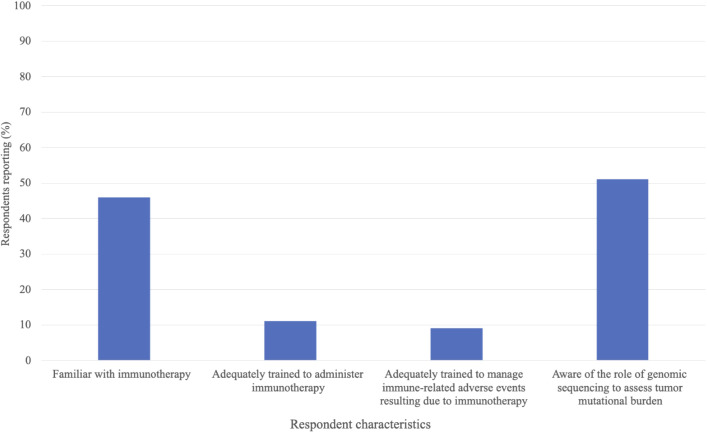
IImmunotherapy knowledge and training in all survey respondents; n=292.

**Figure 3** describes the familiarity with, and training in, immunotherapy for all 292 respondents. 46% of respondents were familiar with immunotherapy. 11% reported being adequately trained to administer it, and 9% reported being adequately trained to manage immune-related adverse results due to immunotherapy administration. 51% of respondents were aware of the role of genomic sequencing to assess tumor mutational burden. 33 respondents indicated being adequately trained to administer immunotherapy. Of these respondents, 52% were also adequately trained to manage immune-related adverse events resulting due to immunotherapy administration, and 79% were aware of the role of genomic sequencing to assess tumor mutational burden (**Table 4**).

**Table 4:** Immunotherapy knowledge and adverse events training in respondents reporting adequate training to administer immunotherapy; n = 33.

Respondent Characteristics	n (%)
**Adequately trained to manage immune-related adverse events resulting due to immunotherapy ** **Aware of the role of genomic sequencing to assess tumor mutational burden**	17 (52) 26 (79)

## 4 Discussion

The dramatic rise of cancer cases and deaths in SSA calls for an assessment of the current cancer treatment landscape in the region, to identify gaps and areas for innovation or improvement. In this study we explored the current utilization of cancer immunotherapy in SSA. To our knowledge, this is the first study to assess the familiarity of, training in, and utilization of, cancer immunotherapy in SSA. Our findings revealed that the use of immunotherapy is limited in SSA clinics, with less than half of respondents’ clinics practicing immunotherapy, and even fewer having adequate resources to administer immunotherapy or easy access to immunotherapy drugs. We believe that the utilization of immunotherapy in SSA is low, or inadequate, for the cancer burden the region faces (1). Limited access to immunotherapy drugs and infusion tools is likely a major barrier to more extensive immunotherapy utilization in the region. At clinics where immunotherapy was practiced, only 12% used genomic sequencing to assess the tumor mutational burden biomarker, and only 44% assessed the PD-L1 biomarker, suggesting limited prediction of immunotherapy response prior to administration. This may decrease the effectiveness and safety of the treatment, leaving more patients at risk for unnecessary autoimmune side effects. While there are additional biomarkers we did not assess in this survey, this finding likely reflects the dearth of trained pathologists and molecular testing facilities within SSA (5, 35, 36). Widespread availability of tumor biomarker testing may be a prerequisite for increased utilization of immunotherapy in SSA.

The most common types of immunotherapy available for administration at respondents’ clinics were monoclonal antibodies and checkpoint inhibitors, which are similarly the most common types of immunotherapy used in some Western countries (37). Our survey results also showed that the most common primary cancers targeted by immunotherapy in this study population were breast cancer, lymphoma, lung cancer, liver cancer and prostate cancer, which suggests the use of immunotherapy drugs for similar cancer types in this population as in the United States (38).

46% of all surveyed healthcare providers were familiar with immunotherapy, 11% reported being trained to administer it, and 9% reported being trained to manage immune-related adverse events induced by immunotherapy. The small proportion of respondents indicating being trained to safely administer immunotherapy is likely partly due to the nature of the clinic positions represented in the survey. Not all survey respondents were healthcare providers typically involved in directly administering treatment. Of respondents reporting being adequately trained to administer immunotherapy, only 52% were also trained to manage immune-related adverse events post treatment. This implies that additional training may be needed in this subgroup to ensure safe administration of immunotherapy and thorough post-treatment monitoring (39). At the end of the survey, respondents were given the opportunity to provide any information they deemed important regarding immunotherapy in an open-response format. A common thread was the current lack of, but desire for, immunotherapy-specific medical training.

Twenty-eight SSA countries were represented in this study, but of 292 responses, 40% were from Nigeria and 11% from Kenya. Nigeria accounts for about 18% of the total population in the SSA region, and Kenya for about 5% of the total population in the region (40). Still, both countries were overrepresented in this survey, which can likely be attributed to our survey distribution method. Nigerian and Kenyan healthcare providers make up about 44% and 10%, respectively, of the online SSA networks of the HypoAfrica clinical trial and BIO Ventures for Global Health to which our survey was distributed. Future research drawing additional survey responses from other global health networks may be worthwhile for a more representative demographic spread.

A noteworthy barrier to more widespread use of immunotherapy in SSA clinics is cost. In many SSA countries, immunotherapy is only accessible to patients who can afford the high cost of the treatment (41). Health insurance coverage is uncommon in this population (42). Due to the ability of immunotherapy to combat advanced stage cancers, which most SSA cancer patients present with (22), efforts should be made to increase the accessibility of immunotherapy in the SSA setting. Public health interventions should be implemented to increase cancer screening and reduce disease progression prior to presentation, but simultaneously, treatments effective against advanced cancer should be promoted to slow the rapidly rising cancer-related mortality in SSA (6). Recent studies have found that ultra-low doses of immunotherapy drugs can increase survival for advanced cancer in low- and middle-income countries. One randomized control trial in India found the addition of an ultra-low dose of an immunotherapy drug to an existing chemotherapy regimen to significantly improve overall survival in patients with advanced head and neck squamous cell carcinoma (43, 44). The dose used in this study was 6% of the standard used for this indication in Western countries, increasing its accessibility, affordability, and cost-effectiveness. Patients may also benefit from the introduction of immunotherapy clinical trials in SSA that make the treatment more accessible.

There are some limitations of our study that are important for us to note. First, our data were based on self-report and employed a self-selected sample, which may have introduced response bias and self-selection bias, respectively. Second, the cross-sectional nature of our survey prevented us from observing changes over time and limited the conclusions we could draw from the data. Additionally, we only assessed the utilization of two immunotherapy biomarkers - tumor mutational burden and PD-L1. While these two biomarkers are the most widely used worldwide, some clinics may utilize others (45, 46). Our survey was only available online in English, which likely restricted responses from healthcare providers without internet access and/or in French-speaking countries, limiting the generalizability of this study. Our survey was also not validated by prior studies before use, however, it was reviewed and modified by oncologists from the United States and Nigeria prior to its distribution for this study, to strengthen its ability to broadly assess immunotherapy availability and practice in SSA. We suggest that future research examine the feasibility and efficacy of introducing immunotherapy training into continuous medical education programs in SSA, and the effectiveness of more widespread use of immunotherapy-specific biomarker testing in SSA clinical settings. Cancer treatment in SSA is often based on clinical trials with Western study populations, though patients in SSA may have a different clinical profile and treatment outcomes (47). Cancer immunotherapy clinical trials should also be conducted with SSA patient populations to assess the efficacy of immunotherapy and the feasibility of more widespread utilization of immunotherapy in the SSA setting, and to encourage international oncology research collaborations.

## 5 Conclusion

This study was the first to evaluate the utilization of cancer immunotherapy in SSA clinical settings. Less than half of the survey respondents indicated that their clinic currently utilizes immunotherapy, and even fewer indicated use of pathologic biomarker testing to predict immunotherapy response. Of healthcare providers reporting being sufficiently trained to administer immunotherapy, only half were trained to manage severe autoimmune side events after immunotherapy administration. Immunotherapy may be a powerful asset to curtail the rapidly increasing cancer mortality in SSA, however, its utilization in the region, and provider training for its safe administration, is insufficient. Prerequisites for wider, safer and effective adoption of cancer immunotherapy in the region may be implementation of immunotherapy and adverse events specific training into continuous medical education, increased clinic access to immunotherapy-related biomarker testing, and increased patient access to immunotherapy drugs. Future research should examine the feasibility and value of introducing immunotherapy training into continuous medical education programs in SSA, and the effectiveness of more widespread use of immunotherapy and immunotherapy-specific biomarker testing for combatting the increasing cancer burden in the region.

## Data availability statement

The raw data supporting the conclusions of this article will be made available by the authors, without undue reservation.

## Author contributions

EO: Formal analysis, Investigation, Methodology, Writing – original draft, Writing – review & editing. SP: Investigation, Writing – review & editing. KG: Resources, Writing – review & editing. AJ: Supervision, Writing – review & editing. NL: Supervision, Writing – review & editing. AM: Resources, Writing – review & editing. CC: Supervision, Writing – review & editing. EJ: Supervision, Writing – review & editing. WN: Conceptualization, Funding acquisition, Supervision, Writing – review & editing.
