# Supplementary material for: Utilization of cancer immunotherapy in sub-Saharan Africa
Source: Front Oncol. 2023 Dec 21;13:1266514. doi: 10.3389/fonc.2023.1266514 (PMC10765613; doi:10.3389/fonc.2023.1266514)
Supplement: Supplementary file 1 [file DataSheet_1.docx]

Supplementary Material

Supplementary Material 1. Immunotherapy survey

INTRODUCTION

The recent Lancet Oncology Commission for sub-Saharan Africa (https://www.thelancet.com/journals/lanonc/article/PIIS1470-2045(21)00720-8/fulltext) highlights the growing cancer crisis in Africa that needs to be urgently addressed in part by increasing access to treatment. The goal of this survey research is to evaluate access to radiotherapy and immunotherapy in Africa. It is designed for health professionals in clinical oncology and radiation oncology. The results will be used to advance efforts to make treatment more accessible in order to address the growing cancer incidence and mortality in various African countries. All responses will remain confidential.

Information including your name, email address, and institute, will be documented for quality assurance of the survey integrity. However, this information will be anonymized during data analysis and publication. Responding to this survey is estimated to take about 15 minutes or less. There are no anticipated risks other than the inconvenience of time spent to complete the survey. There are no direct benefits to you from participating. However, the results may be used to inform the development of strategies and investments to increase access to treatment in Africa. You can skip any questions and stop the survey at any time. Data collected will be used for academic research purposes.

If you have any questions about the survey, contact William_Swanson@student.uml.edu. If you have questions about your rights as a participant, concerns or complaint contact the IRB@uml.edu and reference IRB#: 22-087-NGW-EXM. Thank you for agreeing to help with this needs assessment.

Please enter your email below. You may print a copy of this consent form for your records. Providing your email address indicates that:

• You have read and understand the above information

• You voluntarily agree to participate in the research

• You are 18 years of age or older

Email:

Name:

Institution:

Where is your clinic located (state, country)? ________________________________________

What type of facility is your clinic?

1. Government owned
2. Tertiary
3. Private
4. Other (please specify): ________________________________________

What is your position in the clinic? (select all that apply)

1. Clinical oncologist
2. Radiation oncologist
3. Medical oncologist
4. Surgical oncologist
5. Medical Physicist
6. Nurse
7. Other (please specify): ________________________________________

Does your clinic practice immunotherapy?

1. Yes
2. No
3. Unsure

Describe your familiarity with immunotherapy

1. I am familiar with immunotherapy
2. I am NOT familiar with immunotherapy
3. I am NOT familiar with immunotherapy and I am interested in learning more

What is your capability to administer immunotherapy to patients?

1. I am adequately trained to appropriately administer immunotherapy to my patients
2. I need additional training to be able to administer immunotherapy to my patients
3. I am not sure that I am adequately trained

Are you trained to manage immune-related adverse events (e.g., autoimmune conditions) that may result from providing a patient with cancer immunotherapy?

1. I am trained to manage immune-related adverse events that may result from providing a patient with cancer immunotherapy
2. I need additional training to be able to manage immune-related adverse events that may result from providing a patient with cancer immunotherapy
3. I am not sure that I am adequately trained

If applicable, for which cancer sites does your clinic perform treatment using immunotherapy? (select all that apply)

1. Head and Neck Cancers
2. Spinal Cancer
3. Breast Cancer
4. Lung Cancer
5. Liver Cancer
6. Pancreatic Cancer
7. Prostate Cancer
8. Uterine Cancer
9. Cervical Cancer
10. Rectal Cancer
11. Bladder Cancer
12. Soft Tissue Sarcoma
13. Lymphoma
14. Leukemia
15. None of the above
16. Unsure
17. Other (please specify): ________________________________________

Are you aware of the role of genomic sequencing to assess tumor mutational burden?

1. Yes
2. No
3. Unsure

***Can*** your clinic use genomic sequencing to assess the tumor mutational burden for each patient (i.e., within your clinic's facilities)?

1. Yes
2. No
3. Unsure

***Does*** your clinic use genomic sequencing to assess the tumor mutational burden for each patient (i.e., either within your clinic or by sending samples to another site)?

1. Yes
2. No
3. Unsure

***Can*** your clinic assess patient expression of pathologic biomarker PD-L1 using tissue biopsy (i.e., within your clinic's facilities)?

1. Yes
2. No
3. Unsure

***Does*** your clinic assess patient expression of pathologic biomarker PD-L1 using tissue biopsy (i.e., either within your clinic or by sending samples to another site)?

1. Yes
2. No
3. Unsure

If you answered “No” to the previous question: how do you assess this prior to prescribing immunotherapy? _______________________________________________________________

_____________________________________________________________________________

Does your clinic have the necessary tools to administer immunotherapy (i.e., storage, formulation, infusion tools)?

1. Yes
2. No
3. Unsure

Does your clinic have easy access to immunotherapy through drug companies, independent providers, the healthcare system or the government?

1. Yes
2. No
3. Unsure

If applicable, which types of immunotherapy does your clinic have available for administration?

1. Monoclonal antibodies
2. Checkpoint inhibitors
3. Cytokines
4. Cancer vaccines
5. CAR T-cell therapy
6. None of the above
7. Unsure
8. Other (please specify): ________________________________________

Are you interested in participating in immunotherapy research including clinical trials?

1. Yes
2. No
3. Unsure

Are you interested in participating in clinical trials considering combined radiotherapy and immunotherapy treatment?

1. Yes
2. No
3. Unsure

Have you had any experience administering clinical trials?

1. Yes
2. No
3. Unsure

If you answered “Yes" to the previous question, briefly describe your role and experience:

__________________________________________________________________________________________________________________________________________________________________________________________________________________________________________

Please provide any additional information to clarify any answers above, or that you deem important to share regarding immunotherapy.

__________________________________________________________________________________________________________________________________________________________________________________________________________________________________________
